# Supplementary material for: MetaRibo-Seq measures translation in microbiomes
Source: Nat Commun. 2020 Jun 29;11:3268. doi: 10.1038/s41467-020-17081-z (PMC7324362; doi:10.1038/s41467-020-17081-z)
Supplement: Supplementary file 10 — Supplementary Data 7 [file 41467_2020_17081_MOESM10_ESM.zip › File2/Confidence_High_Taxonomy/31449_out.krona.html]

Javascript must be enabled to view this page.

members
magnitude
magnitudeUnassigned
count
unassigned
taxon
rank

31449\_out

3

3
2
superkingdom

phylum
3
1239

class
186801
3

186802
3
order

species
1

SRS011302\_contig\_number\_17479
1897045

186803
2
family
1

SRS143598\_contig\_number\_14292

841

SRS013098\_contig\_number\_29036
1
genus
